# Therapeutic efficacy of Qingfei Paidu decoction combined with antiviral drugs in the treatment of corona virus disease 2019: A protocol for systematic review and meta analysis

**DOI:** 10.1097/MD.0000000000020489

**Published:** 2020-05-29

**Authors:** Kai Gao, Yan-Ping Song, Hao Chen, Lin-Tao Zhao, Li Ma

**Affiliations:** a Pharmacy College, Shaanxi University of Chinese Medicine, Xianyang; b Shaanxi Academy of Traditional Chinese Medicine, Xi’an, Shaanxi, China.

**Keywords:** antiviral drugs, corona virus disease 2019, meta-analysis, protocol, Qingfei Paidu decoction, systematic review

## Abstract

**Background:**

The corona virus disease 2019 (COVID-19) has caused a global pandemic, there are no specific drugs and vaccines for epidemic control at present. More and more clinical practice shows that traditional Chinese medicine has played an important role in the outbreak. Among them, Qingfei Paidu decoction (QPD) combined with antiviral drugs can enhance the therapeutic efficacy for COVID-19. However, there is still a lack of comprehensive and systematic evidence, which urgently requires us to verify its therapeutic efficacy. Hence, we provide a protocol for systematic review and meta-analysis.

**Methods:**

We will search the studies in MEDLINE/PubMed, China National Knowledge Infrastructure, Wanfang database, VIP database, the Cochrane Library, Chinese Biomedical Database and Chinese Science Citation Database. Searches are limited to clinical studies published in Chinese and English. Next, the quality of each study is assessed according to the criteria of the Cochrane Handbook for Systematic Reviews of Interventions. Then, the outcome data are recorded and pooled by Review Manager 5.3 and STATA 16.0 software.

**Results:**

The systematic review and meta-analysis aims to review and pool current clinical outcomes of QPD combined with antiviral drugs for the treatment of COVID-19.

**Conclusion:**

This study will provide a high-quality evidence of QPD for the treatment on COVID-19 patients.

**PROSPERO Registration Number:**

CRD42020182409.

## 1 Introduction

Since December 2019, the sudden epidemic of corona virus disease 2019 (COVID-19) has seriously threatened the healthy life of the people. As of April 25, 2020, there have been approximately 2,840,000 confirmed cases of COVID-19 worldwide, with 200,000 deaths. Among them, a total of 84,330 cases of COVID-19 (including overseas imported cases) were diagnosed in China, with 78,384 cured cases and 4642 deaths. From these data, it is not difficult to find that China's prevention and control of COVID-19 epidemic is beginning to bear fruit, which is not only due to measures such as restricting the flow of people and vigorously publicizing, but also to the important factor of medical assistance. In particular, the widespread use of Traditional Chinese Medicine (TCM) has played a huge role in the prevention and control of this epidemic.^[1,2]^

During the epidemic, the National Health Commission of the People's Republic of China have formulated and issued “the Diagnosis and Treatment Protocol for Novel Coronavirus Pneumonia (Trial Version)” based on clinical manifestations and pathology of the disease, as well as accumulated experience in diagnosis and treatment.^[3]^ Among them, Qingfei Paidu decoction (QPD) was included the treatment protocol as a recommended prescription. Its scope of application includes light, moderate, and severe patients, and it can be used reasonably in combination with the actual situation of patients in the treatment of critically ill patients. The prescription consists of Mahuang (Ephedrae Herba), Zhigancao (Glycyrrhizae Radix et Rhizoma Praeparata cum Melle), Xingren (Armeniacae Semen Amarum), Shengshigao (Gypsum Fibrosum), Guizhi (Cinnamomi Ramulus), Zexie (Alismatis Rhizoma), Zhuling (Polyporus), Baizhu (Atractylodis Macrocephalae Rhizoma), Fuling (Poria), Chaihu (Bupleuri Radix), Huangqin (Scutellariae Radix), Jiangbanxia (Pinelliae Rhizoma Praeparatum cum Zingibere et Alumine), Shengjiang (Zingiberis Rhizoma Recens), Ziwan (Asteris Radix et Rhizoma), Donghua (Farfarae Flos), Shegan (Belamcandae Rhizoma), Xixin (Asari Radix et Rhizoma), Shanyao (Dioscoreae Rhizoma), Zhishi (Aurantii Fructus Immaturus), Chenpi (Citri Reticulatae Pericarpium), and Huoxiang (Pogostemonis Herba). QPD has been proved to have a significant therapeutic effect on COVID-19, the effective cure rate of QPD for COVID-19 is more than 90%.^[4]^ However, there is still a lack of comprehensive and systematic evidence. Therefore, we will present a meta-analysis protocol of the therapeutic efficacy of QPD combined with antiviral drugs vs antiviral drugs alone on COVID-19.

## 2 Materials and methods

The study protocol has been registered on International prospective register of systematic reviews (PROSPERO ID: CRD42020182409). The protocol followed Preferred Reporting Items for Systematic review and Meta-Analysis Protocols (PRISMA-P) guidelines.^[5]^

### 2.1 Data resources and search strategies

We will search the studies in MEDLINE/PubMed, China National Knowledge Infrastructure, Wanfang database, VIP database, the Cochrane Library, Chinese Biomedical Database, and Chinese Science Citation Database. Searches are limited to clinical studies published in Chinese and English. The target databases are searched for capturing all potentially relevant clinical literature by 2 reviewers independently and disagreements are settled by discussion with a third reviewer.

The following search strategies will be used to identify publications: ((Qingfei Paidu Decoction [Title/Abstract]) AND (((((((((((Antiviral Agents [Title/Abstract]) OR (Agents, Antiviral [Title/Abstract])) OR (Antivirals [Title/Abstract])) OR (Antiviral Drugs [Title/Abstract])) OR (Drugs, Antiviral [Title/Abstract])) OR (α-Interferon [Title/Abstract])) OR (Lopinavir [Title/Abstract])) OR (Ritonavir [Title/Abstract])) OR (Ribavirin [Title/Abstract])) OR (Chloroquine phosphate [Title/Abstract])) OR (Arbidol [Title/Abstract]))) AND ((((((((((((COVID-19 [Title/Abstract]) OR (2019 novel coronavirus disease [Title/Abstract])) OR (COVID19 [Title/Abstract])) OR (COVID-19 pandemic [Title/Abstract])) OR (SARS-CoV-2 infection [Title/Abstract])) OR (COVID-19 virus disease [Title/Abstract])) OR (2019 novel coronavirus infection [Title/Abstract])) OR (2019-nCoV infection [Title/Abstract])) OR (coronavirus disease 2019 [Title/Abstract])) OR (coronavirus disease-19 [Title/Abstract])) OR (2019-nCoV disease [Title/Abstract])) OR (COVID-19 virus infection [Title/Abstract])).

### 2.2 Inclusion and exclusion criteria

The inclusion criteria were designed as following:

(1) The clinical trials involved were randomized controlled trials.
(2) Patients diagnosed with COVID-19 by the following criteria: “the Diagnosis and Treatment Protocol for Novel Coronavirus Pneumonia (Trial Version)” was formulated and issued by the National Health Commission of the People's Republic of China.
(3) The patients in the experimental group received antiviral-based therapy with QPD, whereas patients in the control group were treated with antiviral-based therapy only. Here, antiviral therapy was defined as the administration of antiviral chemical drugs, such as α-interferon, Lopinavir, Ritonavir, Ribavirin, Chloroquine phosphate, Arbidol, etc. In addition, 2 groups of patients can be given circulatory support, effective oxygen therapy, and other support therapies.
(4) The measurement indicators of clinical studies should include one of the following indicators at least: clinical efficacy, relief time of main symptoms (such as fever, cough, and lung CT improved), hematology index (such as the levels of lymphocyte, C-reactive protein, and albumin), and adverse reactions.

We also set exclusion criteria as following:

(1) The patients were only suspected cases of COVID-19 (not confirmed cases).
(2) Articles of the following types should be excluded: comments, non-clinical experiments, self-control studies, case reports, random method error studies, and reviews.
(3) If there were repetitively published clinical literatures, only the latest publications with large sample sizes and more comprehensive studies were included.
(4) If the treatment of COVID-19 patients involved other TCM prescriptions, TCM patent prescriptions, or acupuncture and moxibustion, it should not be included.

**Figure 1:**
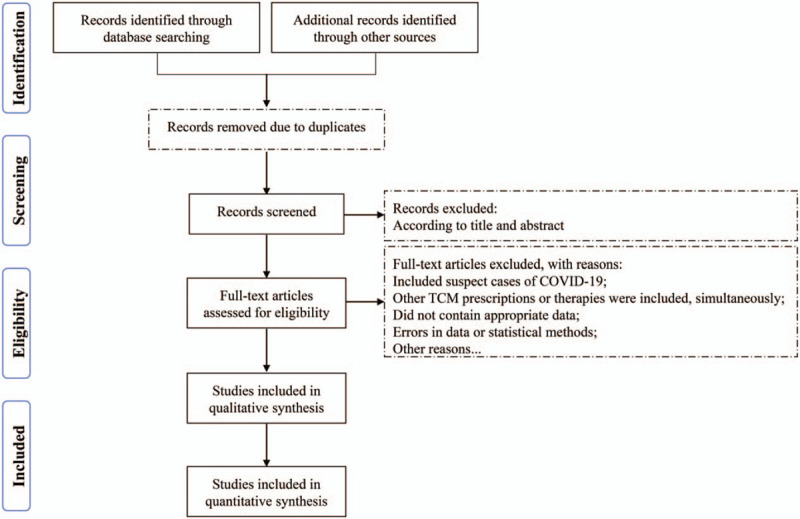
Flow chart for screening qualified studies.

The PRISMA flow chart showed the full screening process (Fig. 1).

### 2.3 Quality assessment of included studies

Two investigators will evaluate independently the methodological quality and bias risk in each trial using “Assessment of Study Quality” in Cochrane Handbook for Systematic Reviews of Interventions,^[6]^ which includes random sequence generation, allocation concealment, blinding of participants and personnel, blinding of outcome assessment, incomplete outcome data, selective reporting, and other bias. If there is a disagreement during the evaluation process, it will be resolved through discussion with a third investigator.

### 2.4 Data collection and analysis

In the process, 2 investigators will extract detailed information and available data from the qualified studies, such as sample size, interventions, duration of intervention, and outcome measures. If there are disagreements during the evaluation process, it will be resolved through discussion with a third investigator. All analyses will be performed with Review Manager 5.3 and STATA 16.0 software.

We will select appropriate statistical methods for meta-analysis according to the data types of each indicator. The dichotomous variables are expressed as a risk ratio (RR) with 95% confidence intervals (95% CI), while the continuous variables are expressed as a mean difference (MD) with 95% CI. Heterogeneity tests are performed by Cochrane's homogeneity test, while *I*^2^ tests are used to quantify the degree of heterogeneity. When *I*^2^ ≤ 25%, the data is considered to be homogeneous. When 25% < *I*^2^ ≤ 50%, the data has lower heterogeneity, and a fixed effect model is used; when *I*^2^ > 50%, the data has obviously heterogeneity, and a random effects model is used.^[7]^ We also seek possible sources of heterogeneity, and attempt to clarify the causes of heterogeneity through subgroup analysis. The publication bias is explored graphically by funnel plots, and detected by Egger test and Harbord test.

## 3 Discussion

Due to the highly contagious and limited medical resources, the epidemic has caused high mortality worldwide, and it has been declared a public health emergency of international concern by the World Health Organization.^[8,9]^ Although the existing antiviral drugs have a certain effect, they still cannot be used as special drugs anti-COVID-19,^[10]^ prompting the need for novel treatment options.

However, TCM could effectively alleviate the development of the epidemic due to its own unique advantages. TCM has the characteristics of overall regulation and the treatment is based on syndrome differentiation. For thousands of years, TCM has formed a unique set of theories, diagnostic, and therapeutic systems as the significant means of treating diseases clinically in China. TCM prescription is a material carrier based on the basic theory of TCM to treat diseases. To achieve the treatment effects of pestilence, it is important to balance yin and yang, promote the body resistance, and eliminate pathogenic factors through a multi-component-target-pathway approach.^[11]^ Also, the network pharmacology results showed that the active ingredients from QPD could contribute to recovery of different disease progresses during COVID-19.^[12]^ Through the implementation of this study protocol, we can comprehensively evaluate the efficacy and safety of QPD in patients with COVID-19, thereby providing reasonable complementary therapy for the clinical treatment of COVID-19.

## Author contributions

**Methodology:** Kai Gao.

**Project administration:** Kai Gao.

**Software:** Hao Chen, Lin-Tao Zhao.

**Supervision:** Yan-Ping Song.

**Visualization:** Lin-Tao Zhao, Li Ma.

**Writing – original draft:** Kai Gao, Hao Chen, Li Ma.

**Writing – review & editing:** Yan-Ping Song.
